# The automatic activity of abdominal muscles during stable and unstable standing postural tasks in older adults with and without low back pain- A cross-sectional study

**DOI:** 10.1186/s12877-024-04934-1

**Published:** 2024-04-02

**Authors:** Mohammad Kalantari, Shabnam ShahAli, Mehdi Dadgoo, Abbas Tabatabaei

**Affiliations:** Iranian Center of Excellence in Physiotherapy, Rehabilitation Research Center, Department of Physiotherapy, School of Rehabilitation Sciences, Iran University of Medical Sciences, Tehran, Iran

**Keywords:** Low back pain, Ultrasonography, Standing postural task, Automatic activity, Abdominal muscles, Postural control

## Abstract

**Background:**

The postural control and abdominal muscles’ automatic activity were found to be impaired in subjects with low back pain (LBP) during static activities. However, the studies are predominantly conducted on younger adults and a limited number of studies have evaluated abdominal muscles’ automatic activity during dynamic standing activities in subjects with LBP. The present study investigated the automatic activity of abdominal muscles during stable and unstable standing postural tasks in older adults with and without LBP.

**Methods:**

Twenty subjects with and 20 subjects without LBP were included. The thickness of the transversus abdominis (TrA), internal oblique (IO), and external oblique (EO) muscles was measured during rest (in supine), static, and dynamic standing postural tasks. To estimate automatic muscle activity, each muscle’s thickness during a standing task was normalized to its thickness during the rest. Standing postural tasks were performed using the Biodex Balance System.

**Results:**

The mixed-model analysis of variance revealed that task dynamicity significantly affected thickness change only in the TrA muscle (*P* = 0.02), but the main effect for the group and the interaction were not significantly different (*P* > 0.05). There were no significant main effects of the group, task dynamicity, or their interaction for the IO and EO muscles (*P* > 0.05). During dynamic standing, only the TrA muscle in the control group showed greater thickness changes than during the static standing task (*P* < 0.05).

**Conclusions:**

Standing on a dynamic level increased the automatic activity of the TrA muscle in participants without LBP compared to standing on a static level. Further research is required to investigate the effects of TrA muscle training during standing on dynamic surfaces for the treatment of older adults with LBP.

## Introduction

Over the last decade, human lifespans have increased significantly, and by 2050, the world population over 60 years old is expected to triple [1, 2]. A rapidly aging population increases the likelihood of non-communicable diseases such as musculoskeletal disorders. The prevalence of musculoskeletal disorders among older adults ranges from 65 to 85% [1, 3], with 36 to 70% suffering from low back pain (LBP) [4, 5].

Previous studies have demonstrated the importance of trunk muscles for spinal stability and their role in the prevention and rehabilitation of LBP [6, 7]. A decrease in trunk muscle efficiency increases the load on the lumbar discs and ligaments, leaving the lumbar region more susceptible to injury and instability [8]. Evidence also suggests that trunk muscles play a critical role in dynamic control of posture [7, 9]. There is a causal link between trunk muscle activity, LBP, and physical function among older adults [10, 11]. Due to these interrelationships and the desire to prevent/treat LBP in this group, trunk muscle activity assessments are needed for older adults.

Abdominal muscles’ automatic activity is sustained tonic activity of these muscles that occurs during a task that loads the spine and pelvis and is considered a protective mechanism for the lumbar spine [12]. Previous studies reported different patterns of abdominal muscles’ automatic activity in subjects with LBP compared to healthy individuals [13–16]. In this regard, one study reported higher automatic activity of superficial abdominal muscle (external oblique (EO)) than deep abdominal muscles (transverse abdominis (TrA), internal oblique (IO)) in subjects with LBP as compared to healthy individuals during standing tasks [15]. Some other studies compared the automatic activity of abdominal muscles between subjects with and without LBP during various tasks and reported a significant difference between groups in abdominal muscles’ automatic activity [17–19].

Studies on the trunk muscles’ automatic activity have been conducted predominantly on younger adults with LBP [13, 15–17]. A healthy aging process is associated with changes in muscle morphology [20]. This age-related change in muscle morphology can impact skeletal muscle activity [21], so, the generalizability of the findings on young people to older adults is questionable. Moreover, a limited number of studies have examined the automatic activity of the abdominal muscles during dynamic tasks.

Daily activities frequently involve dynamic tasks such as standing on unstable surfaces, walking, and climbing stairs that may cause pain in subjects with LBP [22]. One of the final goals of rehabilitation in subjects with LBP is controlling their dynamic posture and pain during daily standing activities. It will enable them to increase their functional capacity [19] gradually. It is unclear whether dynamic postural tasks affect abdominal muscles’ automatic activity in older adults with and without LBP. An evaluation of the effects of standing on unstable surfaces on the automatic activity of superficial and deep abdominal muscles in older adults with and without LBP could provide useful clinical information. This study investigated the automatic activity of superficial and deep abdominal muscles in older adults with and without LBP during stable and unstable standing postural tasks.

## Method

### Study design

This was an observational, cross-sectional study with a two-factor mixed design (two groups × two postural tasks). The human ethical committee of the Iran University of Medical Sciences approved the study (IR.IUMS.REC.1400.265) and participants gave written informed consent to participate in the study. Data collection was performed between March and December 2022.

### Participants

Twenty men with LBP and twenty asymptomatic subjects over 60 years old were recruited. In terms of absolute thickness, males’ abdominal muscles are significantly thicker than females’ [12]. This study examined only males to ensure the homogeneity of the data and better judgment about abdominal muscles thickness changes during postural tasks. Older adults with LBP were recruited from Iran University orthopedic and/or physiotherapy outpatient clinics. Older adults with a history of LBP for more than 3 months, recurrent LBP with at least two episodes that lasted two consecutive days during the last year [23], and a pain score between 0 and 30 mm (mild pain) on a Visual Analog Scale (VAS) on the testing day were included. VAS measures the intensity of pain, and patients rate pain intensity on a scale of 0 (no pain) to 100 mm (worst imaginable pain) [24].

Older adults without LBP (control group) were recruited through advertisements in the University and the local community. The inclusion criteria for the control group were no LBP in the previous year or back pain lasting more than one week in the previous year [25]. The control and LBP groups were matched based on demographic characteristics.

Exclusion criteria for both groups were: history of foot, knee, and hip disorders, pelvic or spinal surgery, congenital spinal malformation or scoliosis, degenerative neurologic disease, severe labyrinthitis; chronic cardiovascular or respiratory diseases, taking medication for pain in the week before the assessment, falls in the past year, Mini-Mental State Examination score < 21 and the Oswestry Disability Index scores between 20 and 60% (moderate to severe disability) [25].

### Standing postural tasks

Static and dynamic standing postural tasks were created through the use of the Biodex Balance System (950 − 304 System, Japan). The Biodex Balance System provides valid and reliable measures of a participant’s ability to maintain balance on stable and unstable surfaces. The platform stability ranges from 1 to 12, with 12 representing the lowest and 1 the greatest instability level [26]. Considering the age of participants and preventing them from losing balance or aggravating LBP symptoms, the bilateral stance at the static level and dynamic level of 8 was used for test postural tasks.

### Ultrasonography

A Diagnostic Ultrasound Imaging (US) system (SONOACE R7, SAMSUNG MEDISON, Korea) set in B-mode with a bandwidth frequency of 6–9 MHz (General frequency), and a linear head transducer was used to measure the thickness of the TrA, IO, and EO muscles. A change in muscle thickness measured with US imaging during a dynamic task could indicate muscle activity [27, 28]. A professionally trained physiotherapist who was blind to the groups of subjects conducted the ultrasound examination.

For the evaluation of the resting thickness of EO, IO, and TrA muscles, subjects were positioned in a supine position (because other positions such as quiet standing, require some muscle activation) [15, 29]. The US transducer was transversely positioned across the right side of the abdominal wall over the anterior axillary line, midway between the 12th rib and the anterior superior iliac crest, allowing a clear image of all three lateral abdominal muscles (TrA, IO, and EO). The distance between the deep and superficial fascia was measured to determine muscle thickness. Analysis was based on average values from the three images [12].

The participants were asked to stand barefoot on the Biodex Balance System platform with arms crossed behind their backs. They were instructed to control their balance without holding the handrails. US transducer motion during dynamic test conditions may distort the images, thus causing errors [30]. Therefore, a transducer fixator was used during standing postural tasks to minimize motion artifacts and improve US reliability [31]. High-density foam was used to make the transducer fixator, which was mounted on an elastic belt [15, 32]. A standard US gel was used during the US assessment either with or without a transducer fixator. Each standing postural task was held enough until the examiner had a clear image of the muscle thickness at the end of expiration, usually no more than 2 min [33]. Abdominal muscles thickness was measured at rest, and while standing on a static level and level 8 of the Biodex Balance System. The measurements were taken on the right side of the abdominal wall with a five-minute interval between trials.

The thickness of each abdominal muscle during postural tasks was expressed as a percentage of that at resting supine position (thickness during postural task/ thickness at rest × 100) [34].

To determine within-day and intra-rater reliability, the ultrasound measurements in both standing postural tasks were repeated twice within a session on 19 participants (9 with LBP, 10 without LBP). The tests were performed in the physiotherapy department of the Iran University of Medical Sciences laboratory in a single session.

### Data analysis

The sample size was calculated based on pilot data collected from 10 subjects in each group, using the G*Power software (version 3.1.9.4) and considering the TrA thickness while standing on an unstable surface. From a priori analysis, a power of 0.8, α = 0.05, and an effect size of 0.46 were set. The sample size of 20 in each group was calculated.

SPSS version 24 was used for analyses. The Shapiro–Wilk test was conducted to evaluate the normality of the distribution of tested variables. In both groups, variables had a normal distribution. Relative reliability was assessed using the two-way mixed model of intra-class correlation coefficients (ICCs).

The association of resting muscle thickness with muscle thickness during standing on dynamic and static levels of the Biodex was represented by data points on scatterplots.

To assess the main effects of the dynamicity of task (2 standing postural tasks (static level, and level 8)) and group (2 health status (LBP, without LBP)), and their interaction effects on the thickness change of abdominal muscles, the 2 × 2 mixed-design analysis of variance (ANOVA) was used. Statistical significance was set at *P* = 0.05 and a post-hoc (Bonferroni) analysis for pairwise comparisons was used to analyze significant main effects and interactions.

## Results

The demographic data are summarized in Table 1. There were no significant differences in age, height, weight, and BMI between groups (*P* > 0.05).

**Table 1:** Demographic data of the subjects in each group (mean (SD))

Variable	Without LBP (*n* = 20)	With LBP (*n* = 20)	P-value
Age (years)	65.20 (5.15)	66.75 (5.78)	0.37
Body mass (kg)	73.25 (6.82)	74.6 (8.94)	0.62
Stature (cm)	170.25 (4.63)	169.35 (6.85)	0.59
BMI (kg/m^2^)	25.32 (2.62)	25.96 (2.25)	0.41
Pain (mm)	-	2.10 (0.78)	-

BMI: body mass index, cm = centimeter, Kg = kilogram, kg/m2: kg is a person’s weight in kilograms and m2 is their stature in meters squared, LBP: low back pain, mm = millimeter, SD: standard deviation

In general, there was excellent within-day reliability for US measurements of abdominal muscle thickness in both groups, during both standing postural tasks. The within-day ICCs ranged from 0.95 to 0.99 in LBP and 0.96 to 0.99 in without LBP groups, respectively.

**Fig. 1:**
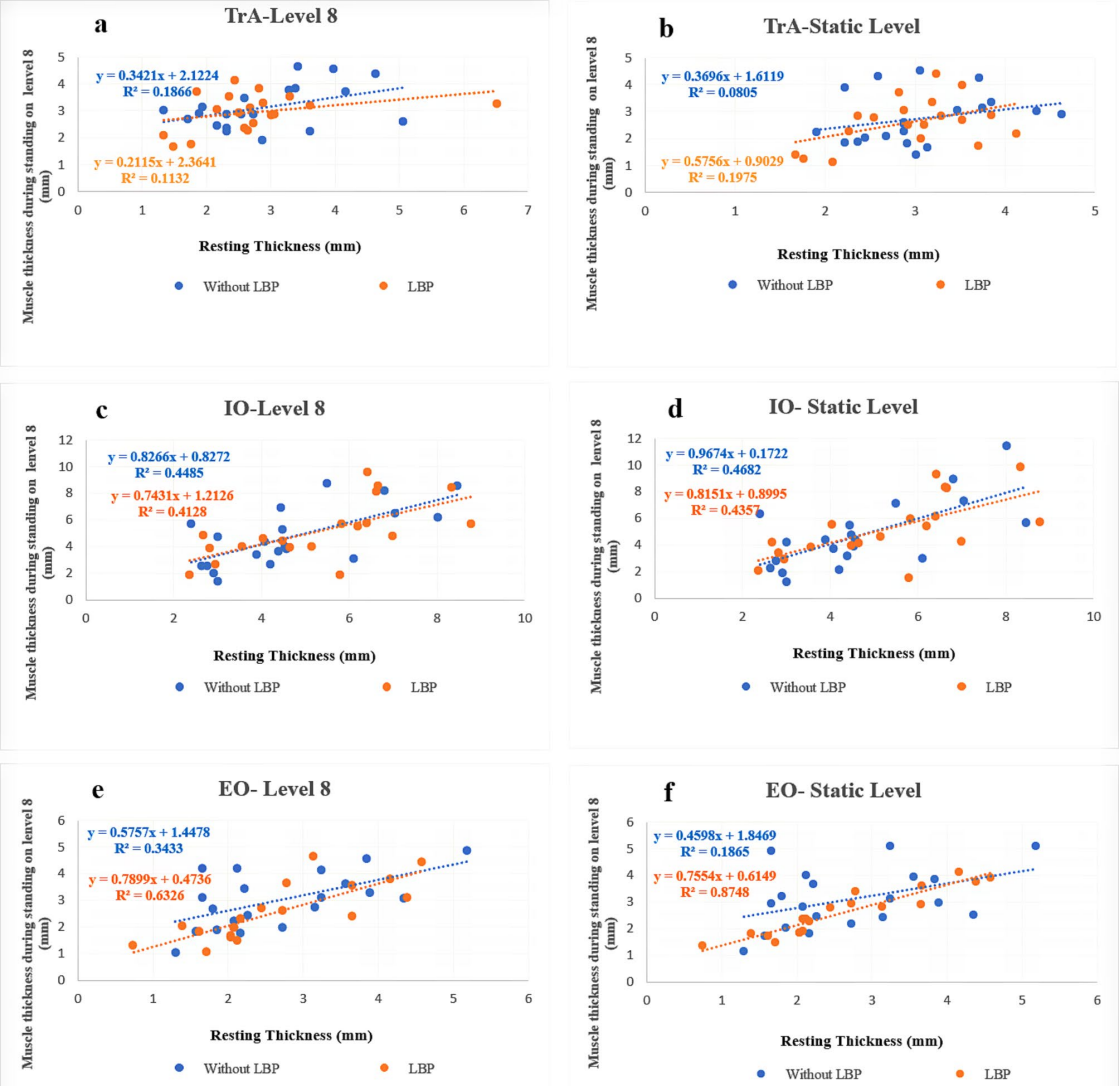
Scatter plots for: **(a)** TrA muscle thickness while standing on level 8 of the Biodex and TrA resting thickness in the supine position. **(b)** TrA muscle thickness while standing on a static level of the Biodex and TrA resting thickness in the supine position. **(c)** IO muscle thickness while standing on level 8 of the Biodex and IO resting thickness in the supine position. **(d)** IO muscle thickness while standing on a static level of the Biodex and IO resting thickness in the supine position. **(e)** EO muscle thickness while standing on level 8 of the Biodex and EO resting thickness in the supine position. **(f)** EO muscle thickness while standing on a static level of the Biodex and EO resting thickness in the supine position. EO: external oblique, IO: internal oblique, LBP: low back pain, mm: millimeters, TrA: transverse abdominis

Scatter plot graphs with the abdominal muscle’s resting thickness in the supine position and abdominal muscles’ thickness while standing on dynamic and static levels of the Biodex are presented in Fig. 1. The R square values for the association of resting muscle thickness with muscle thickness during standing position on a dynamic level of Biodex ranged from 0.11 to 0.63 in LBP and 0.18 to 0.44 in control groups. For the association of resting muscle thickness with muscle thickness during standing on a static level of the Biodex, R square values ranged from 0.19 to 0.87 and 0.08 to 0.46 in LBP and control groups respectively.

The results of 2 × 2 mixed-design ANOVA (Table 2) indicate the significant main effect of task dynamicity on the thickness change of only the TrA muscle (*P* = 0.02), but the main effect for health status (group) and the interaction were not significantly different (*P* > 0.05). There were no significant main effects of health status (group), task dynamicity, or their interaction for the IO and EO muscles (*P* > 0.05).

**Table 2:** Results of the 2 × 2 mixed-design ANOVA (percentage of changes in the thickness of abdominal muscles during two standing postural tasks in elderly subjects with and without low back pain)

Muscle	Task			Group			Task×Group		
	F	P-value	Partial eta squared	F	P-value	Partial eta squared	F	P-value	Partial eta squared
TrA	5.28	0.02*	0.12	0.00	0.93	0.00	1.59	0.21	0.04
IO	0.44	0.50	0.01	0.00	0.93	0.00	1.10	0.30	0.02
EO	0.97	0.33	0.02	3.61	0.06	0.08	0.54	0.46	0.14

EO: external oblique, IO: internal oblique, TrA: transverse abdominis

The percentage of thickness changes of each muscle between two standing tasks in each group is shown in Table 3. Only the TrA muscle in the control group demonstrated significantly greater automatic activity in standing on level 8 than standing on a static level (*P* = 0.03).

**Table 3:** Pair-wise comparison of the percentage of changes in the thickness of abdominal muscles between postural tasks in elderly subjects with and without low back pain

Muscle	Group	Task	Task	Mean difference (95% CI)	*P*- value	Effect size
TrA	Without LBP	1	2	-7.47 (-14.29; -0.66)	0.03*	0.21
	With LBP	1	2	-2.17 (-7.72; 3.38)	0.42	0.06
IO	Without LBP	1	2	-0.3.93 (-12.03; 4.16)	0.32	0.08
	With LBP	1	2	0.88 (-4.25; 6.01)	0.72	0.02
EO	Without LBP	1	2	0.71 (-9.41; 10.83)	0.88	0.01
	With LBP	1	2	4.95 (-1.56; 11.47)	0.12	0.17

CI: confidence interval, EO: external oblique, IO: internal oblique, LBP: low back pain, TrA: transverse abdominis, Task 1: standing on a static level, Task 2: standing on level 8 (dynamic level

## Discussion

In this study, the automatic activity of the abdominal muscles during stable and unstable standing postural tasks was assessed in older adults with and without LBP. According to the results, there was a main effect of task dynamicity for the TrA muscle. Standing on a dynamic level significantly increased TrA muscle automatic activity compared to standing on the static level in the control group.

Multiple factors are involved in maintaining postural control or balance. Previous studies have investigated several aspects of postural control during standing in older adults including muscle strength, force [35, 36], and endurance [37]. Since maintaining postural control relies on constant position correction via muscle activity [38, 39], examining muscle activity in dynamic positions is important. Abdominal muscles are widely believed to assist in spinal stabilization [40, 41]. It is believed that the automatic activation of these muscles is a protective mechanism for the lumbar spine when the stability level decreases [16, 18].

Typically, electromyographic measurements are used to describe muscle activity, such as the onset and amplitude of electrical excitation. In order to accurately measure deep abdominal muscle activity using electromyography, fine-wire electrodes must be inserted into the muscles. Subjects usually experience discomfort during this process, especially if they have to move [42, 43]. Several studies have used US imaging to measure changes in muscle thickness to indirectly assess muscle activity in recent years [44–46]. Compared to intramuscular EMG recordings, US is noninvasive and could be used clinically. Moreover, it allows for an examination of a larger area of the muscle than is possible with a single intramuscular electrode [47, 48].

The results of the present study showed that only the TrA muscle was significantly affected by task dynamicity. In the control group, while standing on a dynamic level, TrA, the deepest abdominal muscle [49], appears more active than when standing on a static level. This finding supports the hypothesis that dynamic standing tasks can increase the activity of deep abdominal muscles more than static standing tasks [15, 29]. However, the task dynamicity did not significantly affect the IO and EO muscles’ automatic activity. This is contrary to the findings of a previous study that found significantly greater activation of all three abdominal muscles during standing on a dynamic surface as compared to static standing tasks in young adults with and without LBP [15]. The reason for the difference in the findings could be due to the age of the participants. The results of a study investigating age-related changes in the thickness of the deep and superficial abdominal muscles showed that loss of muscle thickness may occur earlier in the IO and EO muscles than in the TrA muscle [20]. In the present study, standing on a dynamic level, increased the TrA muscle automatic activity compared to standing on a static level, only in older adults without LBP. In contrast, other studies reported more TrA muscle automatic activity on dynamic surfaces than on static surfaces among participants with and without LBP [18, 20]. Age-related changes in muscle quantity and quality might explain these differences in findings. Although the thickness loss is lower in the TrA as a result of aging, the actual contractile tissue in this muscle has decreased in comparison with young people due to an augmentation of fat and connective tissue [20]. Even though both groups were probably affected by the age-related changes in muscle morphology, the difference in the response of the TrA muscle to task dynamicity may be explained by other factors, such as pain or changes in neuromuscular activation in the LBP group [50–52].

The increased automatic activity of the TrA muscle in the control group, when the postural control challenge is increased, is consistent with the theory of an increased postural role for TrA during more challenging postures [53]. The automatic activity of the TrA muscle has been proposed as an indicator of deep abdominal muscles’ ability to increase lumbar stability [54, 55]. Improving lumbar stability is an important step in the treatment of LBP [56]. Accordingly, some researchers advocate unstable training in rehabilitation to improve deep abdominal muscle activity and increase lumbar stability [57, 58]. It is recommended that future studies investigate the effects of TrA muscle training while standing on dynamic surfaces for the treatment of older adults with LBP.

The findings of this study revealed that most subjects in both groups demonstrated a linear positive correlation between the resting thickness of the IO and EO muscles and the thickness of these muscles during standing tasks. However, there was a poor correlation between the resting thickness of the TrA muscle and its thickness during standing tasks in both groups. In other words, the TrA muscle thickness during standing tasks was unpredictable based on its resting thickness, which may allow flexible activation of this muscle to exploit its mechanical properties and to adapt movement to environmental perturbations [59].

The results of the present study differed from those observed in young adults that indicated standing on unstable surfaces compared to standing on stable surfaces significantly increases the automatic activity of TrA, IO, and EO muscles in both with and without LBP groups [15, 18]. Inherent age-related factors such as sarcopenia and changes in motor control possibly influenced the findings of this study.

## Limitations

In this study, only old men with mild pain were recruited. So, the results cannot be generalized to the older female population or old men with higher levels of pain intensity. Further studies on the effects of task dynamicity on abdominal muscles’ automatic activity in old women, middle-aged men, and women with higher levels of pain intensity are suggested.

Also, it is suggested that future research compares the effects of unstable standing on other aspects of muscle activity such as muscle timing variables (e.g., onset, latency), in older adults with and without LBP. Moreover, this study did not assess the participants’ physical activity levels, so there may have been differences between the groups. Future studies should consider this factor. Despite these limitations, these findings provide some clues to future research on motor control exercise in rehabilitation programs for older adults with LBP.

## Conclusion

Based on the results of the study, the automatic activity of the TrA muscle in participants without LBP increases during standing on the dynamic level compared to standing on the static level. This finding suggests that standing on a dynamic level of Biodex can increase the demand on the TrA muscle to maintain trunk posture in older adults without LBP. Future research is needed to examine the effects of TrA muscle training during standing on dynamic surfaces for the treatment of older adults with LBP.

## Data Availability

The data presented in this study are available on reasonable request from the corresponding author.

## Abbreviations

ANOVA: Analysis of variance
EO: External oblique
ICC: intra-class correlation coefficient
IO: Internal oblique
LBP: Low back pain
TrA: Transversus abdominis
VAS: Visual analog scale
US: Ultrasound Imaging
